# Laparoscopic surgery on broken points for uterine sarcoma in the early stage decrease prognosis

**DOI:** 10.1038/srep31229

**Published:** 2016-08-09

**Authors:** Hong Liu, Yi Zhu, Guo-Nan Zhang, Chang Wang, Chao Li, Yu Shi

**Affiliations:** 1 Department of Gynecologic Oncology, Sichuan Cancer Hospital, Chengdu, Sichuan, PR China; 2 Department of Ultrasound, Sichuan Cancer Hospital, Chengdu, Sichuan, PR China; 3 Department of Obstetrics and Gynecology, Chengdu First People’s Hospital, Chengdu, Sichuan, PR China; 4 Department of Obstetrics and Gynecology, Chengdu Second People’s Hospital, Chengdu, Sichuan, PR China

## Abstract

Uterine sarcoma, a rare solid tumor in uterus, is difficult to identify in the early stage from some benign uterine tumors, such as uterine fibroids. Hence, uterine sarcoma may be treated in the same way as uterine fibroids; and this may not be found until pathological diagnosis. Consequently, this can lead to tumor’s abdominal spread, planting and local invasive growth, resulting in an early uterine sarcoma, an increased relapse rate after surgery and a decreased survival. Therefore, it’s important to avoid these unintended and iatrogenic complications through an accurate diagnosis and an appropriate surgical approach. The surgical staging and a complete resection of the tumor are both important for patients’ prognosis. In this review, we will discuss the laparoscopic surgery for uterine sarcoma in the early stage and patients’ prognosis.

Uterine sarcoma is a rare solid tumor in uterus accounting for ~1% of gynecological tumors and ~3% of uterine malignancies. Approximately 0.13–0.81% of uterine sarcoma is derived from benign uterine leiomyoma^1^. The major histological types of uterine sarcoma consist of uterine leiomyosarcoma (LMS) (accounting for ~40% of all uterine sarcomas), endometrial stromal sarcoma (ESS) (10–15%), (high grade) undifferentiated sarcoma (UES) (5%)^2^ and other pure heterologous sarcomas. Because of hematogenous metastasis, direct spread and planting within the abdominal cavity and an early occurrence of lymphatic metastasis, the prognosis is poor without surgery^1^,^2^. Prognosis of uterine sarcoma is associated with the tumor stage and the performance of a complete tumor resection^3^. Uterine sarcoma usually occurs in women at an age of between 50 and 60 years^4^. Because clinical manifestation of uterine sarcoma is not characteristic in the early stage, it is difficult to diagnose it from some benign uterine tumors, such as uterine fibroids. Hence, a surgical approach to uterine fibroids can be adopted to treat uterine sarcoma. It is until a postoperative pathological examination that uterine sarcoma can be accurately diagnosed. Hysterectomy or uterine fibroid resection, known as uterine surgery sub broken (uterine morcellation) or tumor sub broken surgery (tumor morcellation), requires the use of high-speed rotation of the electric sub-broken device after peeling and removing the uterus or fibroids from the abdominal cavity. If laparoscopic surgery in benign tumors is mistakenly used in uterine sarcoma, the electric sub-broken tissues may facilitate abdominal spread. Thus, diagnosis of uterine sarcoma from benign tumors is of significance in choosing an appropriate surgery approach, which is crucial for patents’ prognosis.

## Accurate diagnosis of uterine sarcoma in the early stage

### Rapid growth of uterine tumor

Although uterine sarcoma in the early stage (stage I or II) does not have characteristics in clinical manifestation, the phenomenon “womb (tumor) rapid growth” has been found in some patients^5^,^6^,^7^,^8^. Such phenomenon may be a valuable clue in the early diagnosis of uterine sarcoma. Researchers found that uterine fibroid may triple the size within 3–6 months^9^; however it is controversial to define “rapid growth” because only 0.27–2.6% patients with “womb rapid growth” were confirmed as LMS^10^.

Patients with uterine fibroid tend to be younger than those with uterine sarcoma (Table 1). In perimenopausal or postmenopausal women with uterine fibroid, the estrogen levels are usually very low. The uterine fibroids in these women should not continue to grow. Therefore, tumor with “womb (tumor) rapid growth” is highly likely to be uterine sarcoma.

### Coagulation necrosis of tumor tissue

Pathology is a gold standard to diagnose uterine sarcoma. The histopathological characteristics of uterine sarcoma include tumor coagulation necrosis, atypical cells with moderate to severe nuclear division (usually 5–10/HPF), and rare inflammatory cells. The coagulation necrosis is a key characteristic in pathologically differential diagnosis between uterine sarcoma and uterine fibroid that usually appears in uterine sarcoma^11^.

Meanwhile, ultrasound or MRI scans of uterine sarcoma have shown tumor degeneration and necrosis^12^,^13^, which is consistent with the pathological characteristics. Uterine fibroids with an increasing necrosis should be suspected as uterine sarcoma as fast-growth of tumor may lead to local ischemia and necrosis. In scanning soft tissue, MRI is more suitable than CT to obtain morphological information. MRI T1-weighted images of uterine sarcoma show a large heterogeneous muscle invasive with low-density, whereas with a medium to high signal intensity on T2-weighted images. In contrast, T2-weighted images usually have low signal intensity in uterine fibroids^14^,^15^,^16^.

In summary, uterine fibroids have a high incidence among perimenopausal and menopausal women. Fast-growth of tumor leads to coagulation necrosis, which can be a clue of early screen in uterine sarcoma. Based on characteristics of coagulation necrosis, diagnostic curettage is a sensitive method to screen ESS, which has been used to diagnose over 60% of ESS^17^.

## Early surgical treatment of uterine sarcoma

Surgery is a major and an effective treatment of uterine sarcoma in the early stage^5^,^7^,^8^ compared with radiotherapy and chemotherapy^18^,^19^,^20^,^21^,^22^,^23^. It usually cuts off uterus and bilateral salpingo to ensure a complete resection of sarcoma. For patients in the early stage, it is controversial whether to keep the ovaries or to do a pelvic lymphadenectomy. However, for menopausal patients in the early stage, this may not be associated with patients’ prognosis^24^,^25^. The rate of ovarian metastasis in patients with early LMS is 3.4–3.9%^26^,^27^,^28^,^29^ and the preservation of ovarian tissues may not increase the risk of recurrence^30^,^31^,^32^,^33^,^34^,^35^,^36^. The rate of lymph node metastasis in LMS patients in the early stage is 0–3.7%^37^,^38^,^39^,^40^. Therefore, it’s not recommended to dissect lymph node in LMS patients in the early stage. ESS is an estrogen-related tumor and it’s recommend to do an early excision of bilateral annex even for premenopausal patients. However, recent studies have shown that ovarian retention does not affect the prognosis of the premenopausal patients in the early stage^41^,^42^,^43^,^44^. Patients who kept ovaries did not increase the incidence of recurrence^8^,^24^,^25^. A study with 50 patients with low-grade endometrial stromal sarcoma showed that postoperative abdominal relapse rate was not associated with preserved ovaries^8^. Therefore, premenopausal patients with ESS in the early stage could choose to retain ovarian tissue according to individual counseling. In the early ESS, the rate of lymph node metastasis is 0-5%^24^,^25^,^34^,^38^,^40^. Hence, it’s not recommended for those patients to take lymphadenectomy.

Laparoscopic hysterectomy and myomectomy are surgical approaches to treat uterine fibroids, in which the fibroid or uterine is taken by motorized broken handles. However, uterine fibroid without pathological diagnosis is possible to be uterine sarcoma. Once uterine sarcoma is broken as uterine fibroid, malignant tumors may spread, resulting in a bad or fatal outcome^5^,^8^,^11^,^45^,^46^,^47^,^48^.

Although it’s not reported any factors on misdiagnosis of surgery carved pieces, the field of version during the surgery may affect the judgment of tumor, and the necrotic cells in pathological examine may help to make a diagnosis^11^. These factors are determinate in selection of a surgical approach for patients and the extent of cutting off.

## Uterus morcellation and metastasis of uterine sarcoma

It’s uterus morcellation that the uterus is broken and removed from abdominal in laparoscopic surgery. Sub-process requires the use of high-speed rotation of broken electric sub and a broken filter through sub-peeling uterus (tumor). When treat uterine sarcoma in a laparoscopic approach used for uterine fibroids, malignant tissues may be broken and spread in abdominal and pelvic, result in metastasis^49^. Recurrent abdominal paracentesis or operate hole may increase the risk of tumor metastasis^11^,^46^. LMS metastasis has a higher incidence than other uterine malignancies. The fact that uterine sarcoma is unpredictable in surgery possibly leads to uterus morcellation, which is a bad or even a fatal outcome^11^,^45^,^46^. A retrospective study with 123 patients with uterine sarcoma showed that they were misdiagnosed as benign tumors and underwent laparoscopic surgery^50^. Among them, 34 cases (28%) incurred uterus morcellation, which may increase the risk of postoperative metastasis within three months. Recently, other studies also showed that laparoscopic surgery may lead to malignant planting in operating hole, abdominal and pelvic sarcoma^11^,^49^,^51^,^52^,^53^.

The residual tissue fragments after sarcoma sub broken operation may cause uterine sarcoma lesions to develop in the early stage. Einstein *et al*.^54^ reported eight cases of uterine sarcoma in the early stage. The patients underwent a complete and an initial staging surgery, and were followed up by 6–61 months. Five cases were on stage I LMS and underwent sub surgery with tumor broken. Of these five cases, four underwent a complete surgical staging again. Two of the three patients who underwent surgery using sub broken were progressed to stage III (abdominal pelvic metastases). Three cases of the early ESS underwent abdominal surgery, and no one got progression before the secondary surgery. Forty percent of patients who underwent surgery with sub broken incurred the tumor, which may be due to tumor metastasis from broken and residual tumor tissue fragments after operation. Della *et al*.^55^ reported a preoperative diagnosis uterine leiomyoma in a 30-year-old patient, who underwent laparoscopic hysterectomy. However, the pathological diagnosis was LGESS. Although no residual tumor was left, the tumor was recurrent, and even more, sarcoma widely disseminated in abdominal and invaded the ovaries and fallopian tubes. These findings suggested that uterus morcellation may lead to tumor abdominal dissemination and metastasis.

## Uterine sarcoma sub broken surgery increases the risk of tumor recurrence and metastasis

Due to a high recurrence of uterine sarcoma despite the surgery in the early stage, the five-year survival is low even after chemotherapy. The recurrence rate of the early LMS is up to 71%^56^. Among these, 40% have lung metastasis^57^. It’s approximately 25–35% of the early ESS develops recurrence, which most usually leads to metastasis in abdominal, followed by vaginal and lung^58^. However, Park *et al*.^8^ reported that among 50 patients with LGESS, abdominal ranked fourth of tumor recurrence and metastasis. In any case, abdominal tended to be suffered in tumor recurrence and metastasis.

In the early stage, such as stage I and II, uterine sarcoma is limited in uterus or uterine without metastasis. However, the tumor may soon spread into abdominal or pelvic, the key body parts for tumor treatment. Broken surgery is generally considered to sub-induce recurrence of early uterine sarcoma, mainly caused by the spreading of sarcoma tissues into peritoneum and abdominal organs during the surgery. Even by going through the operating holes, sub broken surgery may cause abdominal and pelvic sarcoma metastasis, resulting in a rapid development of this tumor. The early uterine sarcoma may not limit in the uterus as it is broken into pieces during the surgery that are easily to plant in womb. In 1991, First Semm proposed the hysterectomy with manually broken and in 1993, Setiner applied the hysterectomy with broken electric devices in clinical. Setiner improved this technique to be more powerful and safer. During the surgery, the tumor was pulverized by a rotary blade when rotate at high speed peeling, in which a few tumor fragments may drop into abdominal, result in tumor spread and metastasis after operation. Moreover, when tumor tissues are squeezed and broken, tumor cells may locally infiltrate and then lead to recurrence in local or cervical stump. This recurrence may be associated with tumor squeezing during surgery^50^. These may partly account for a higher incidence of recurrence after sub broken surgery than that after open operation^5^,^8^,^55^,^59^. Park *et al*.^5^ found that patients with LMS after sub-groups broken surgery had a higher rate of tumor metastasis and recurrence in vaginal stump during early postoperative period, suggesting an association between the surgery approach and tumor recurrence. Moreover, tissue broken during surgery may increase the risk of misdiagnosis as it is difficult to target samples.

## Impact on the quality of life after surgery treatment for early uterine sarcoma

If the early uterine sarcoma was misdiagnosed as benign tumor, the tumor issue may be broken during the surgery. This may lead to sarcoma planting, dissemination and metastasis, and finally reducing survival of patients even in the early stage^5^,^8^.

Park *et al*.^5^ found that in addition to tumor stage, sub broken surgery may influence the overall survival (OS) of postoperative patients with LMS. Furthermore, patients who did not undertake broken surgery are more likely to reserve ovarian (38.7% vs72%, P = 0.013).

Aside from LMS, LGESS patients’ prognosis is affected by the surgery. The 5-year disease-free survival (DFS) of patients without sub-broken surgery was significantly higher than these underwent the surgery^8^. Moreover, the time before recurrence was longer in patients without sub-broken surgery than those with the surgery. However, the OS of LGESS patients was not found to be associated with the sub-broken surgery, which may be due to the inactively biological characteristics of LGESS.

## Preventive measures

Sub broken surgery is usually used to treat uterine fibroids. However, if being used to cut off uterine sarcoma, tumor may disseminate and plant in abdominal. Therefore, an accurate diagnosis of uterine sarcoma is significant to choose an appropriate approach and to avoid the unintended and iatrogenic tumor spread.

Uterine sarcoma surgery requires a complete removal of the tumor and uterus. The tumor size may influence the early postoperative survival of patients with uterine sarcoma^5^,^60^. However, laparoscopic surgery has no specific requirements on the tumor size. For example, some studies showed that uterine fibroids with a size of 6 cm–10 cm should not be operated by laparoscopic surgery^61^,^62^ and the others reported that when the width of fundus or lower uterine segment by CT scan is larger than 8 cm, it should not be cut by minimally invasive surgery^63^. It has been reported that the average size of uterus in LMS and LGESS was 8.6 cm 5 and 5.9 cm 8, respectively, indicating that the size of uterus in LGESS patients may be smaller. Hence it is difficult to select suitable patients to do laparoscopic surgery based on the size of tumor.

As prognostic points broken surgery used for early uterine sarcoma is divided mainly on broken device, it is essential to improve sub-broken device. Although the technique has been improved since the first report of the sub broken surgery in 1991, the rotating blades broken may still cause uterine sarcoma to spread and plant in abdominal. It may help by replacing the existed rotating blade with the smooth blade with bipolar role^14^. It is recommended to remove all possible tissue fragments by breaking it into a morcellation bag, reducing the chance of tissue residuals. However, this method does not completely remove the residual tumor tissue fragments. If all tumor fragments could be broken and cleaned out in sub-broken surgery under endoscope, the possibility of spreading tumor residues may be reduced^64^. However, this method couldn’t guarantee to clean out all tumor fragments.

It may be helpful to remove tumor fragments more cautiously before the new technique comes out. One way is to repeatedly wash the celiac in order to take out tumor fragments as much as possible. In fact, these measures are necessary to reduce tumor spread not only during the operation on uterine sarcoma but also on adenomyosis, uterine fibroids and tumor in uterine smooth muscle with low malignant potential. These measures can minimize planting tumor fragments in abdominal during the operation^8^,^45^,^65^,^66^,^67^,^68^,^69^,^70^, which may finally lead to iatrogenic and parasitic uterine fibroids^6^,^54^,^71^,^72^,^73^,^74^,^75^.

Supplemented treatment or even secondary cytoreductive surgery may improve prognosis for patients with tumor recurrence caused by sub-broken surgery^76^. Park *et al*.^8^ reported that seven of nine patients with tumor recurrence were improved by supplemented treatment, including chemotherapy and endocrine therapy. For patients with uterine sarcoma in the early stage who underwent surgery with broken line points, both radiotherapy and chemotherapy are important to improve the survival^60^. These patients should be followed up after the treatments to observe the long-term efficacy. For patients with benign uterine disease underwent hysterectomy surgery or sub-broken surgery, it’s difficult to have an accurate pathologic diagnosis. Einstein^54^ found that patients who underwent subtotal hysterectomy or uterine surgery due to misdiagnosis of benign tumor, surgical exploration and re-staging are significant for their prognosis. Patients’ prognosis may be positive if the surgical stage is consistent with the preoperative one. However, the surgical stage of 15% patients is advanced, especially for the LMS patients underwent broken surgery with line points. Thus, surgical stage may be important in prognosis of LMS patients and in selection of treatment strategy. Oduyebo *et al*.^77^ found that among the 21 patients (15 with ULMS and six with smooth muscle tumors of uncertain malignant potential (STUMP) who underwent broken surgery and no metastasis, twelve who had a surgical exploration underwent a reoperation about one month later after the first surgery. Among seven patients, two were on the stage I of UTMS and four were on the stage I of STUMP. They all had an exploration during the operation and found immediately disseminated intra-abdominal lesions. Among these 21 patients, eight patients in the early UTMS and STUMP received a secondary exploratory surgery for peritoneal recurrence. This shows that doing surgical exploration again after broken surgery is beneficial for an accurate assessment of prognosis. These findings suggested that re-exploration in the broken surgery is important for patients’ prognosis.

## Conclusion

Broken surgery on uterine sarcoma may decrease patients’ survival because of tumor spread, planting and metastasis in abdominal. Since uterine fibroid is usually treated by laparoscopic surgery, the differential diagnosis of uterine sarcoma from uterine fibroid has been concerned in order to avoid adverse outcome from the inappropriate surgical approach^6^,^7^. Although the incidence of uterine sarcoma is low, an inappropriate surgical approach may significantly reduce patients’ survival due to tumor residuals. Therefore, surgical approach should be carefully selected when uterine tumors were suspected to be sarcoma ones. Recently, Frumovitz suggested that^64^, patients should be strictly excluded malignant lesions of the uterus and cervix before laparoscopic hysterectomy surgery. Patients should be operated according to the principles of surgical treatment of malignant tumors if they doubt malignancy. When selecting pulverization technique, it’s very important to select patients with indication.

## Additional Information

**How to cite this article**: Liu, H. *et al*. Laparoscopic surgery on broken points for uterine sarcoma in the early stage decrease prognosis. *Sci. Rep.* **6**, 31229; doi: 10.1038/srep31229 (2016).

## Figures and Tables

**Table 1:** Different characteristics of uterine sarcoma and uterine fibroid.

Uterine sarcoma	Uterine fibroid
Rare	Common
Poor prognosis	Good prognosis
Invasive, necrotic, and hemorrhagic	Tend to have a firm, creamy white surface
Usually occur after menopause	Develop primarily of reproductive age
Usually diagnosed at age 50 and older	Usually diagnosed around age 40–50
Cancerous	Benign

## References

[b1] Berchuck A. . Treatment of uterine leiomyosarcoma. Obstet. Gynecol. 71, 845–850 (1988). 2453004

[b2] Tavassoli F. A. DP e. World Health Organization classification of tumours In: Pathology and genetics of tumours of the breast and female genital organs M. Lyon IARC Press (2003).

[b3] Zhang G. Surgery treatment on uterine sarcoma. J. Practical Obste. Gynecol. 28, 11–13 (2012).

[b4] Leibsohn S., d’ Ablaing. G., Mishell D. R. Jr & Schlaerth J. B. Leiomyosarcoma in a series of hysterectomies performed for presumed uterine leiomyomas. Am. J. Obstet. Gynecol. 162, 968–974 (1990). 2327466 10.1016/0002-9378(90)91298-q

[b5] Park J. Y. . The impact of tumor morcellation during surgery on the prognosis of patients with apparently early uterine leiomyosarcoma. Gynecol. Oncol. 122, 255–259 (2011). 21565389 10.1016/j.ygyno.2011.04.021

[b6] Leung F. . Hysterectomies performed for presumed leiomyomas: should the fear of leiomyosarcoma make us apprehend non laparotomic surgical routes? Gynecol. Obstet. Fertil. 37, 109–114 (2009). 19200764 10.1016/j.gyobfe.2008.09.022

[b7] Leung F. & Terzibachian J. J. Re. The impact of tumor morcellation during surgery on the prognosis of patients with apparently early uterine leiomyosarcoma. Gynecol Oncol. 124, 172–173 (2012). 21955481 10.1016/j.ygyno.2011.08.035

[b8] Park J. Y. . The impact of tumor morcellation during surgery on the outcomes of patients with apparently early low-grade endometrial stromal sarcoma of the uterus. Ann. Surg. Oncol. 18, 3453–3461 (2011). 21541824 10.1245/s10434-011-1751-y

[b9] Curtin J. P., Kavanagh J. J., Fox H. & Spanos W. J. Corpus: mesenchymal tumors. In Hoskins W. J., Perex C. A. & Young R. C. (eds). Principles and practice of gynecologic oncology. Philadelphia (PA), Lippincott Williams and Wilkins 919 (2002).

[b10] Parker W. H., Fu Y. S. & Berek J. S. Uterine sarcoma in patients operated on for presumed leiomyoma and rapidly growing leiomyoma. Obstet. Gynecol. 83, 414–418 (1994). 8127535

[b11] Anupama R. . Disseminated peritoneal leiomyosarcomas after laparoscopic “myomectomy” and morcellation. J. Minim. Invasive. Gynecol. 18, 386–389 (2011). 21545964 10.1016/j.jmig.2011.01.014

[b12] Szabo I., Szantho A. & Papp Z. Uterine sarcoma: diagnosis with multiparameter sonographic analysis. Ultrasound. Obstet. Gynecol. 10, 220–221 (1997). 9339532 10.1046/j.1469-0705.1997.10030220.x

[b13] Kido A. . Diffusely enlarged uterus: evaluation with MR imaging. Radiographics 23, 1423–1439 (2003). 14615554 10.1148/rg.236035033

[b14] Tanaka Y. O. . Smooth muscle tumors of uncertain malignant potential and leiomyosarcomas of the uterus: MR findings. J. Magn. Reson. Imaging 20, 998–1007 (2004). 15558559 10.1002/jmri.20207

[b15] Ho K. C. . 18F-fluorodeoxyglucose positron emission tomography in uterine carcinosarcoma. Eur. J. Nucl. Med. Mol. Imaging 35, 484–492 (2008). 17952435 10.1007/s00259-007-0533-z

[b16] Ueda M., Otsuka M., Hatakenaka M. & Torii Y. Uterine endometrial stromal sarcoma located in uterine myometrium: MRI appearance. Eur. Radiol. 10, 780–782 (2000). 10823633 10.1007/s003300051004

[b17] Jin Y. . Clinical characteristics of endometrial stromal sarcoma from an academic medical hospital in China. Int. J. Gynecol. Cancer 20, 1535–1539 (2010). 21370596

[b18] Amant F. . Clinical management of uterine sarcomas. Lancet. Oncol. 10, 1188–1198 (2009). 19959075 10.1016/S1470-2045(09)70226-8

[b19] Nam J. H. & Park J. Y. Update on treatment of uterine sarcoma. Curr. Opin. Obstet. Gynecol. 22, 36–42 (2010). 19923988 10.1097/GCO.0b013e328334d90f

[b20] Park J. Y. . Prognostic factors and treatment outcomes of patients with uterine sarcoma: analysis of 127 patients at a single institution, 1989–2007. J. Cancer. Res. Clin. Oncol. 134, 1277–1287 (2008). 18506484 10.1007/s00432-008-0422-2 PMC12161709

[b21] Benoit L. . The role of surgery and treatment trends in uterine sarcoma. Eur. J. Surg. Oncol. 31, 434–442 (2005). 15837053 10.1016/j.ejso.2005.01.010

[b22] Sagae S. . Preoperative diagnosis and treatment results in 106 patients with uterine sarcoma in Hokkaido, Japan. Oncology 67, 33–39 (2004). 15459493 10.1159/000080283

[b23] Nam E. J. . Endometrial stromal sarcomas: a retrospective analysis of 28 patients, single center experience for 20 years. Cancer. Res. Treat. 40, 6–10 (2008). 19688058 10.4143/crt.2008.40.1.6 PMC2699082

[b24] Shah J. P. . Lymphadenectomy and ovarian preservation in low-grade endometrial stromalsarcoma. Obstet. Gynecol. 112, 1102–1108 (2008). 18978112 10.1097/AOG.0b013e31818aa89a

[b25] Li A. J. . Ovarian preservation in stage I low-grade endometrial stromal sarcomas. Obstet. Gynecol. 106, 1304–1308 (2005). 16319256 10.1097/01.AOG.0000185511.91694.1e

[b26] Major F. J. . Prognostic factors in early-stage uterine sarcoma: a Gynecologic Oncology Group study. Cancer 71 (4 Suppl), 702–709 (1993). 10.1002/cncr.2820710440 8381710

[b27] Geszler G. . Prognostic value of peritoneal washings in patients with malignant mixed mullerian tumors of the uterus. Am. J. Obstet. Gynecol. 155, 83–89 (1986). 3014883 10.1016/0002-9378(86)90084-0

[b28] Leitao M. M. . Incidence of lymph node and ovarian metastases in leiomyosarcoma of the uterus. Gynecol. Oncol. 91, 209–212 (2003). 14529683 10.1016/s0090-8258(03)00478-5

[b29] Gard G. B., Mulvany N. J. & Quinn M. A. Management of uterine leiomyosarcoma in Australia. Aust. N. Z. J. Obstet. Gynaecol. 39, 93–98 (1999). 10099759 10.1111/j.1479-828x.1999.tb03453.x

[b30] Berchuck A. . Treatment of uterine leiomyosarcoma. Obstet. Gynecol. 71, 845–850 (1988). 2453004

[b31] Larson B., Silfversward C., Nilsson B. & Pettersson F. Prognostic factors in uterine leiomyosarcoma. A clinical and histopathological study of 143 cases. The Radiumhemmet series 1936–1981. Acta. Oncol. 29, 185–191 (1990). 2334571 10.3109/02841869009126543

[b32] Van Dinh T. & Woodruff J. D. Leiomyosarcoma of the uterus. Am. J. Obstet. Gynecol. 144, 817–823 (1982). 7148905 10.1016/0002-9378(82)90358-1

[b33] Giuntoli R. 2nd. . Retrospective review of 208 patients with leiomyosarcoma of the uterus prognostic indicators, surgical management, and adjuvant therapy. Gynecol. Oncol. 89, 460–469 (2003). 12798712 10.1016/s0090-8258(03)00137-9

[b34] Gadducci A. . Uterine leiomyosarcoma: analysis of treatment failures and survival. Gynecol. Oncol. 62, 25–32 (1996). 8690287 10.1006/gyno.1996.0185

[b35] Wu T. I. . Prognostic factors and impact of adjuvant chemotherapy for uterine leiomyosarcoma. Gynecol. Oncol. 100, 166–172 (2006). 16182349 10.1016/j.ygyno.2005.08.010

[b36] Soh L. T., Chew S. H. & Ang L. Uterine leiomyosarcoma: a Singapore experience. Aust. N. Z. J. Obstet. Gynaecol. 39, 246–248 (1999). 10755790 10.1111/j.1479-828x.1999.tb03383.x

[b37] Major F. J. . Prognostic factors in early-stage uterine sarcoma: a Gynecologic Oncology Group study. Cancer 71, 1702–1709 (1993). 8381710 10.1002/cncr.2820710440

[b38] Ayhan A. . Uterine sarcoma: the Hacettepe hospital experience of 88 consecutive patients. Eur. J. Gynaecol. Oncol. 18, 146–148 (1997). 9105869

[b39] Kapp D. S., Shin J. Y. & Chan J. K. Prognostic factors and survival in 1396 patients with uterine leiomyosarcomas: emphasis on impact of lymphadenectomy and oophorectomy. Cancer 112, 820–830 (2008). 18189292 10.1002/cncr.23245

[b40] Goff B. A. . Uterine leiomyosarcoma and endometrial stromal sarcoma: lymph node metastases and sites of recurrence. Gynecol. Oncol. 50, 105–109 (1993). 8349151 10.1006/gyno.1993.1172

[b41] Amant F. . Clinical study investigating the role of lymphadenectomy, surgical castration and adjuvant hormonal treatment in endometrial stromal sarcoma. Br. J. Cancer 97, 1194–1199 (2007). 17895898 10.1038/sj.bjc.6603986 PMC2360466

[b42] Chan J. K. . Endometrial stromal sarcoma: a population-based analysis. Br. J. Cancer 99, 1210–1215 (2008). 18813312 10.1038/sj.bjc.6604527 PMC2570503

[b43] Chu M. C. . Low-grade endometrial stromal sarcoma: hormonal aspects. Gynecol. Oncol. 90, 170–176 (2003). 12821359 10.1016/s0090-8258(03)00258-0

[b44] Kim W. Y. . Low-grade endometrial stromal sarcoma: a single center’s experience with 22 cases. Int. J. Gynecol. Cancer 18, 1084–1089 (2008). 18179547 10.1111/j.1525-1438.2007.01159.x

[b45] Larrain D. . “Iatrogenic” parasitic myomas: unusual late complication of laparoscopic morcellation procedures. J. Minim. Invasive. Gynecol. 17, 719–724 (2010). 20655285 10.1016/j.jmig.2010.05.013

[b46] Shan L., Wang Z. & Yue Y. Uterine sarcoma with abdominal wall metastasis following laparoscopy: case report. Eur. J. Gynaecol. Oncol. 32, 590–591 (2011). 22053685

[b47] Lynam S. . Risk, risk reduction and management of occult malignancy diagnosed after uterine morcellation: a commentary. Crit. Rev. Oncol. Hematol. 98, 302–308 (2016). 26673851 10.2217/whe.15.63

[b48] Cui R. R. & Wright J. D. Risk of occult uterine sarcoma in presumed uterine fibroids. Ann. Surg. Oncol. 23, 1501–1507 (2016). 26645385 10.1097/GRF.0000000000000163

[b49] Guyon F. . A critical assessment of morcellation in case of uterine malignancies and its impact on gynecologic surgery: From “precautionary principle” to “realism”. Obstet. Gynecol. 127, 18–22 (2016). 26657189 10.1016/j.bulcan.2015.10.013

[b50] Morice P. . Prognostic value of initial surgical procedure for patients with uterine sarcoma: analysis of 123 patients. Eur. J. Gynaecol. Oncol. 24, 237–240 (2003). 12807231

[b51] Parker W. H. . U.S. food and drug administration’s guidance regarding morcellation of leiomyomas: well-intentioned, but is it harmful for women? Obstet. Gynecol. 127, 29–39 (2016). 26646134 10.1097/AOG.0000000000001157

[b52] Raine-Bennett T. . Occult uterine sarcoma and leiomyosarcoma: incidence of and survival associated with morcellation. Clin. Obstet. Gynecol. 59, 103–118 (2016). 26646120 10.1097/AOG.0000000000001187

[b53] Bogani G. . Morcellation of undiagnosed uterine sarcoma: a critical review. Bull. Cancer 103, 96–103 (2016). 26672915 10.1016/j.critrevonc.2015.11.015

[b54] Einstein M. H. . Management of uterine malignancy found incidentally after supracervical hysterectomy or uterine morcellation for presumed benign disease. Int. J. Gynecol. Cancer 18, 1065–1070 (2008). 17986239 10.1111/j.1525-1438.2007.01126.x

[b55] Della B. C. & Karini H. Endometrial stromal sarcoma diagnosed after uterine morcellation in laparoscopic supracervical hysterectomy. J. Minim. Invasive. Gynecol. 17, 791–793 (2010). 20955991 10.1016/j.jmig.2010.07.001

[b56] Major F. J. . Prognostic factors in early-stage uterine sarcoma. A Gynecologic Oncology Group study. Cancer 71, 1702–1709 (1993). 8381710 10.1002/cncr.2820710440

[b57] D’Angelo E. & Prat J. Uterine sarcomas: a review. Gynecol. Oncol. 116, 131–139 (2010). 19853898 10.1016/j.ygyno.2009.09.023

[b58] Malouf G. G. . Impact of adjuvant treatment modalities on the management of patients with stagesI-II endometrial stromal sarcoma. Ann. Oncol. 21, 2102–2106 (2010). 20305035 10.1093/annonc/mdq064

[b59] Rekha W., Amita M., Sudeep G. & Hemant T. Unexpected complication of uterine myoma morcellation. Aust. N. Z. J. Obstet. Gynaecol. 45, 248–249 (2005). 15904454 10.1111/j.1479-828X.2005.00397.x

[b60] Casali P. G. & Sanfilippo R. Uterine sarcomas: a multidisciplinary challenge. Eur. J. Cancer 47, 326–327 (2011). 21944001 10.1016/S0959-8049(11)70189-7

[b61] Reich H., Thompson K. A., Nataupsky L. G., Grabo T. N. & Sekel L. Laparoscopic myomectomy: an alternative to laparotomy myomectomy or hysterectomy? Gynecol. Endosc. 6, 7–12 (1997).

[b62] Mais V. . Laparoscopic versus abdominal myomectomy: a prospective, randomized trial to evaluate benefits in early outcome. Am. J. Obstet. Gynecol. 174, 654–658 (1996). 8623802 10.1016/s0002-9378(96)70445-3

[b63] Allan Covens R. K. Laparoscopic Surgery for Gynecologic Oncology. M. McGraw-Hill Companies 1–2 (2009).

[b64] Frumovitz M. Laparoscopic total hysterectomy vs supracervical hysterectomy: turn, turn, turn. J. Minim. Invasive. Gynecol. 17, 669–670 (2010). 20955976 10.1016/j.jmig.2010.08.001

[b65] Bisceglia M. . Selected case from the arkadi m. Rywlin international pathology slide series: leiomyomatosis peritonealis disseminata: report of 3 cases with extensive review of the literature. Adv. Anat. Pathol. 21, 201–215 (2014). 24713991 10.1097/PAP.0000000000000024

[b66] Al-Talib A. & Tulandi T. Pathophysiology and possible iatrogenic cause of leiomyomatosis peritonealis disseminata. Gynecol. Obstet. Invest. 69, 239–244 (2010). 20068330 10.1159/000274487

[b67] Miyake T. . A case of disseminated peritoneal leiomyomatosis developing after laparoscope-assisted myomectomy. Gynecol. Obstet. Invest. 67, 96–102 (2009). 18946223 10.1159/000164949

[b68] Takeda A. . Parasitic peritoneal leiomyomatosis diagnosed 6 years after laparoscopic myomectomy with electric tissue morcellation: report of a case and review of the literature. J. Minim. Invasive. Gynecol. 14, 770–775 (2007). 17980343 10.1016/j.jmig.2007.07.004

[b69] Donnez O. . Posthysterectomy pelvic adenomyotic masses observed in 8 cases out of a series of 1405 laparoscopic subtotal hysterectomies. J. Minim. Invasive. Gynecol. 14, 156–160 (2007). 17368249 10.1016/j.jmig.2006.09.008

[b70] Decenzo J. A. Iatrogenic endometriosis caused by uterine morcellation during a supracervical hysterectomy. Obstet. Gynecol. 103, 583–584 (2004). 15024758

[b71] Aust T. 1., Gale P., Cario G. & Robertson G. Bowel resection for iatrogenic parasitic fibroids with preoperative investigations suggestive of malignancy. Fertil. Steril. 96, e1–e3 (2011). 21620392 10.1016/j.fertnstert.2011.04.097

[b72] Sinha R. . Parasitic myoma after morcellation. J. Gynecol. Endosc. Surg. 1, 113–115 (2009). 22442523 10.4103/0974-1216.71612 PMC3304268

[b73] Paul P. G. & Koshy A. K. Multiple peritoneal parasitic myomas after laparoscopic myomectomy and morcellation. Fertil. Steril. 85, 492–493 (2006). 16595233 10.1016/j.fertnstert.2005.10.017

[b74] Rabischong B. . Long-term complication of laparoscopic uterine morcellation: iatrogenic parasitic myomas. J Gynecol. Obstet. Biol. Reprod (Paris). 42, 577–584 (2013). 23973119 10.1016/j.jgyn.2013.07.006

[b75] Epstein J. H., Nejat E. J. & Tsai T. Parasitic myomas after laparoscopic myomectomy: case report. Fertil. Steril. 91, 932, e13–e14 (2009). 18922520 10.1016/j.fertnstert.2008.08.014

[b76] 2^nd^, Giuntoli R. L. . Secondary cytoreduction in the management of recurrent uterine leiomyosarcoma. Gynecol. Oncol. 106, 82–88 (2007). 17434579 10.1016/j.ygyno.2007.02.031

[b77] Oduyebo T. . The value of re-exploration in patients with inadvertently morcellated uterine sarcoma. Gynecol. Oncol. 132, 360–365 (2014). 24296345 10.1016/j.ygyno.2013.11.024

